# Antiproliferative effects of sorafenib and pegylated IFN-α2b on human liver cancer cells *in vitro* and *in vivo*

**DOI:** 10.3892/ijo.2013.1904

**Published:** 2013-04-16

**Authors:** HIRONORI KUSANO, SACHIKO OGASAWARA, JUN AKIBA, MASAMICHI NAKAYAMA, KOSUKE UEDA, HIROHISA YANO

**Affiliations:** Department of Pathology, Kurume University School of Medicine, Kurume, Japan

**Keywords:** hepatocellular carcinoma, pegylated interferon-α2b, sorafenib, combination therapy, microvessel density

## Abstract

Novel therapeutic strategies are needed to treat patients with advanced hepatocellular carcinoma (HCC). Combination therapy of sorafenib and type I interferon (IFN) has substantial activity in patients with metastatic renal cell carcinoma. We investigated the antiproliferative effects of sorafenib in combination with pegylated interferon-α2b (PEG-IFN-α2b) on human hepatocellular carcinoma (HCC) cells *in vitro* and *in vivo*. A poorly differentiated HCC cell line derived from a patient with hepatitis C virus infection, HAK-1B and the moderately differentiated HCC cell line KIM-1 were used in this study. We demonstrated a synergistic antiproli ferative effect of combination therapy on HAK-1B cells *in vitro*. In the *in vivo* study, a significant reduction of tumor volume and weight were observed in the combination group in both HAK-1B and KIM1 tumors, although synergistic effects were not clearly observed. The density of CD34-positive microvessels was significantly lower and cleaved caspase-3-positive apoptotic cell numbers were higher, in the sorafenib group and the combination group compared to the control or PEG-IFN-α2b group in both HAK-1B and KIM-1 tumors. Ki67 labeling index was significantly lower in the combination group compared to the control group in KIM-1 tumors. In conclusion, our results suggest that the combination therapy may be more effective for the treatment of HCC cases with variable sensitivity to antitumor effects of single therapy with either sorafenib or PEG-IFN-α2b.

## Introduction

Primary liver cancer, of which hepatocellular carcinoma (HCC) represents the major subtype accounting for between 85 and 90%, is the sixth most common tumor globally and the third most common cause of cancer-related death (1). Systemic treatment options for advanced HCC are limited and most deaths occur within 1 year of diagnosis (2–4).

Sorafenib is an oral multikinase inhibitor that was approved by the US Food and Drug Administration in December 2005 for the treatment of advanced renal cell carcinoma (RCC) and in November 2007 for the treatment of HCC. It has been shown to inhibit the activity of Raf kinase and several receptor tyrosine kinases, including vascular endothelial growth factor receptors (VEGFR)-1, 2 and 3, platelet-derived growth factor receptor (PDGFR)-α and β, FLT3, Ret and c-Kit. The intracellular signaling pathway Raf/MEK/ERK and the extracellular receptors VEGFR and PDGFR have been implicated in the molecular pathogenesis of HCC (5).

The Sorafenib Hepatocellular Carcinoma Assessment Randomized Protocol (SHARP) trial revealed efficacy of sorafenib in the treatment of HCC, i.e., both median survival and time to progression showed 3-month improvements by sorafenib therapy (6). Cheng *et al*(7) also reported the efficacy of sorafenib in patients in the Asia-Pacific region with advanced hepatocellular carcinoma. Combination therapy with sorafenib has a potential to improve the outcome of sorafenib monotherapy. Phase II trial of combination therapy of sorafenib and IFN-α has substantial activity in patients with metastatic RCC (8,9). The combination therapy of IFN-α and 5-fluorouracil is partly or completely effective in about 50% of the patients with advanced HCC (10). Type I interferon (IFN) has various effects, including anti-viral effects, antiproliferative effects and anti-angiogenic effects (11), and our laboratory previously reported the antiproliferative effect of IFN-α on human liver cancer cells *in vitro* and *in vivo*(12–14). In addition, type I IFN has suppressive effects on the occurrence of HCC, and the recurrence of HCC after curative treatment in patients with chronic hepatitis C virus infection (15–20). On the basis of above-described background, our current study examined the growth inhibitory effects of combination treatment of sorafenib and Pegylated IFN-α2b (PEG-IFN-α2b) on human HCC cell lines *in vitro* and *in vivo*.

## Materials and methods

### Cell line and cell cultures

This study used two HCC cell lines [KIM-1 (21) and HAK-1B (22)], which were originally established and characterized in our laboratory and previously confirmed to retain morphological and functional characteristics of the original tumor. Both of these two cell lines were established from surgically resected HCC nodules. KIM-1 is a moderately differentiated HCC cell line, and HAK-1B is a poorly differentiated HCC cell line which was derived from a patient with hepatitis C virus (HCV) infection.

The cells were grown in Dulbecco’s modified Eagle’s medium (Nissui Seiyaku Co., Tokyo, Japan) supplemented with 2.5% heat-inactivated (56°C, 30 min) fetal bovine serum (FBS, Bioserum, Victoria, Australia), 100 U/ml penicillin, 100 mg/ml streptomycin (Gibco-BRL/Life Technologies Inc., Gaithersburg, MD, USA) and 12 mmol/l sodium bicarbonate, in a humidified atmosphere of 5% CO_2_ in air at 37°C.

### Sorafenib and pegylated IFN-α2b

Sorafenib, kindly provided by Bayer Pharmaceutical Corporation (West Haven, CT, USA), was dissolved in dimethyl sulfoxide (DMSO) to create a 10 mM stock solution and stored at −20°C for *in vitro* study. For the *in vivo* study, we prepared the solution at time of use.

PEG-IFN-α2b (PEG Intron^®^) was kindly provided by MSD K.K. (Tokyo, Japan). The specific activity of PEG-IFN-α2b was 6.4×10^7^ IU/mg protein.

### Effect of sorafenib alone or combination treatment of sorafenib and PEG-IFN-α2b on the proliferation of HCC and CHC cell lines in vitro

The effects of sorafenib and/or PEG-IFN-α2b on the growth of the cultured cells were examined with colorimetry using 3-(4,5-dimethylthiazol-2yl)-2,5-diphenyl tetrazolium bromide (MTT) assay kits (Chemicon International Inc.) as described (12–14). Briefly, the cells (1.5–5.5×10^3^ cells per well) were seeded on 96-well plates (Nunc Inc., Roskilde, Denmark), cultured for 24 h, and the culture medium was changed to a new one containing 0.2% DMSO (control) or sorafenib (0.3125, 0.625, 1.25, 2.5, 5, 10 or 20 *μ*M), or both sorafenib (0, 1.25, 2.5 or 5 *μ*M) and PEG-IFN-α2b (0, 2,000, 4,000, 8,000 IU/ml) (constant-ratio combination). After culturing for 72 h, the number of viable cells was measured with ImmunoMini NJ-2300 (Nalge Nunc International, Tokyo, Japan) by setting the test wavelength at 570 nm and the reference wavelength at 630 nm. To keep the optical density within linear range, all experiments were performed while the cells were in the logarithmic growth phase.

Combination analysis was performed by using the method as described by Chou and Talalay (23), and the CalcuSyn software program (Biosoft, Cambridge, UK) for automated analysis. This program calculates the combination index (CI). A CI of 0.9–1.1 indicates a nearly additive effect, a CI of <0.9 a synergistic effect, a CI of >1.1 an antagonistic effect.

### Morphological observation

For morphological observation under a light microscope, cultured HAK-1B cells were seeded on Lab-Tek tissue culture chamber slides (Nunc Inc.), cultured with or without 1.25 *μ*M of sorafenib for 72 h, fixed for 10 min in Carnoy’s solution, and stained with hematoxylin and eosin (H&E).

### Quantitative analysis of apoptotic cells induced by sorafenib and/or PEG-IFN-α2b

HAK-1B and KIM-1 were cultured with the culture medium containing 0.02% DMSO or 2 *μ*M of sorafenib for 72 h. For a study of combination therapy, HAK-1B cells were cultured with sorafenib (1.25 *μ*M) or PEG-IFN-α2b (2,000 IU/ml), or both sorafenib (1.25 *μ*M) and PEG-IFN-α2b (2,000 IU/ml) for 72 h. After incubation, the cells were stained with the Annexin V-EGFP (enhanced green fluorescent protein) using Apoptosis Detection Kits (Medical and Biological Laboratories, Nagoya, Japan) according to the manufacturer’s protocol. After staining, the cells were analyzed using a FACScan (Becton-Dickinson Immunocytometry Systems, San Jose, CA, USA), and the rate of Annexin V-EGFP-positive apoptotic cells was determined.

### Effects of sorafenib and/or PEG-IFN-α2b on HCC cell proliferation in nude mice

This experiment was approved by the institutional committee for animal experiments and conducted according to the ‘Guide for the Care and Use of Laboratory Animals’ published and revised by the National Institute of Health in 1985.

Cultured HAK-1B or KIM-1 cells (1.0×10^6^ cells/mouse) were transplanted subcutaneously (s.c.) to 4-week-old female BALB/c athymic nude mice (Clea Japan Inc., Osaka, Japan). On the 7th day when tumor size became 5 to 10 mm in diameter (day 0), the mice were divided into four groups (n=8 each) in a manner to equalize the mean tumor diameter of every group. Each group was assigned to one of the four treatments: i) control; ii) PEG-IFN-α2b alone; iii) sorafenib alone; and iv) sorafenib + PEG-IFN-α2b (combination).

Sorafenib was diluted with 12.5% Cremophor EL/12.5% ethanol/75% water for oral dosing in mice. Sorafenib (200 *μ*g/day) was administered by tube feeding once a day for 14 days. PEG-IFN-α2b (1,920 IU) was subcutaneously injected twice a week for 14 days (days 1, 4, 8 and 11). In the control and the sorafenib alone groups, 0.1 ml of medium as the replacement of PEG-IFN-α2b was injected subcutaneously twice a week. In the control and the PEG-IFN-α2b alone groups, 0.2 ml of Cremophor EL/ethanol/water (12.5/12.5/75) as the replacement of sorafenib was administered by tube feeding once a day. The dose of sorafenib (200 *μ*g) in the ratio to the average bodyweight of a mouse (20 g) was 10 mg/kg and this is almost comparable to a clinical dose (800 mg total daily dose). The clinical dose of PEG-IFN-α2b in chronic hepatitis C is 96,000 IU/kg per week. Because of species difference and different target which is not virus, but tumor, we used twice the dose per week in nude mice.

Tumor size was measured in two directions using calipers, and tumor volume (mm^3^) was estimated by using the equation: length × (width)^2^ × 0.5. This measurement was performed every two days. Mouse body weight was measured on days 0, 7 and 14. Mouse was sacrificed and the tumor was resected the next day after the completion of the 14-day treatment (day 15). The resected tumor was fixed in formalin after the weight measurement, prepared into paraffin sections, and underwent HE staining and immunohistochemistry.

### Immunohistochemistry

Paraffin-embedded tissue samples were cut into 4-*μ*m sections. Anti-mouse CD34 (Rat monoclonal, MEC14.7, Abcam, Cambridge, UK) (1:50 dilution) and Ki67 (Rabbit monoclonal, SP6, Abcam, Cambridge, UK) (1:100 dilution) staining were performed by standard avidin-biotin-peroxidase complex method and 3,3′-diaminobenzidine (DAB) solution was used for color development. Cleaved caspase-3 (rabbit polyclonal antibody, Cell Signaling Technologies, Beverly, MA, USA) (1:250 dilution) staining was performed on the Discovery XT automated staining system (Ventana Medical Systems, Tucson, AZ, USA) to detect the apoptotic cells. This automated system uses the streptavidin-biotin complex method with DAB as a chromogen (Ventana iView DAB detection kit).

Microvessel density (MVD) was evaluated within the tumor according to a modified method introduced by Tanigawa *et al*(24). Briefly the slides stained with CD34 were screened at low power field (×40 or ×100) and the two or three most vascular areas were selected. Microvessel counts of these areas were performed at high power field (×200, 0.74 mm^2^). All positive stained cells were counted as microvessels and every 40 *μ*m length of vessel lumen was calculated as one point. The average microvessel counts of selected areas were regarded as MVD, which was expressed as the absolute number of microvessels per 0.74 mm^2^. Immunohistochemically, cleaved caspase-3 was expressed perinuclearly and Ki67 was on the nuclear. The rate of apoptotic cells and Ki67 labeling index were evaluated by calculating the rate of cleaved caspase-3-positive cells and Ki67-positive cells, respectively.

### Statistical analysis

Comparisons of estimated tumor volume and colorimetric cell growth were performed using two-factor factorial ANOVA and Student’s t-test, respectively. The other data comparisons were performed using the Mann-Whitney U test.

## Results

### Effect of sorafenib alone or combination treatment of sorafenib and PEG-IFN-α2b on the proliferation of HAK-1B or KIM-1 HCC cells in vitro

**Figure 1:**
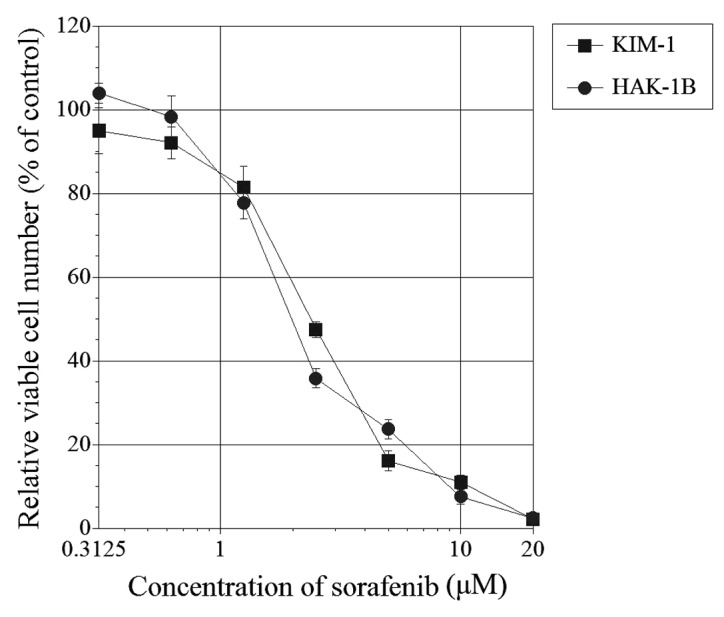
Seventy-two hours after adding 0.3125, 0.625, 1.25, 2.5, 5, 10 or 20 *μ*M of sorafenib. Cell proliferation was suppressed in a dose-dependent manner in both KIM-1 and HAK-1B cell lines. The suppression was significant (P<0.001–0.05) in the range of 0.625–20 *μ*M of sorafenib in KIM-1, 1.25–20 *μ*M in HAK-1B. A total of 50% growth inhibitory dose was 2.5 *μ*M in KIM-1 and 2.1 *μ*M in HAK-1B. The values represent mean ± SD.

Seventy-two hours after the addition of sorafenib, the relative viable cell number was suppressed in both HAK-1B and KIM-1 cell lines in a dose-dependent manner (Fig. 1). The 50% inhibitory concentration (IC_50_) was 2.1 *μ*M for HAK-1B and 2.5 *μ*M for KIM-1.

**Figure 2:**
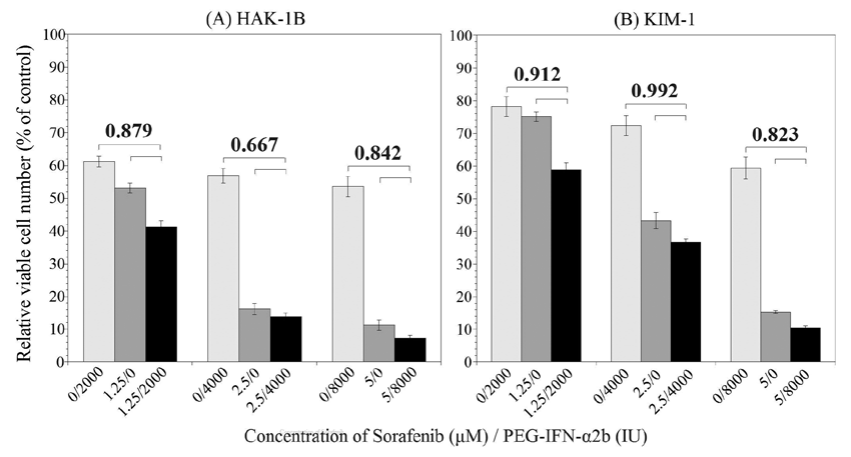
Effect of PEG-IFN-α2b and/or sorafenib on the proliferation of human HCC cell lines (A) HAK-1B and (B) KIM-1 in culture for 72 hours. Light gray bars are PEG-IFN-α2b alone group, dark gray bars sorafenib alone group, and black bars PEG-IFN-α2b + sorafenib group. All combination groups showed significant difference compared with monotherapy groups. The numbers above bars are CI. A CI of 0.9–1.1 indicates a nearly additive effect, a CI of <0.9 a synergistic effect, a CI of >1.1 an antagonistic effect. Representative data of two independent experiments are shown. The values represent mean ± SD.

Seventy-two hours after the addition of PEG-IFN-α2b and sorafenib, the relative viable cell number was suppressed to various degrees. The results are shown in Fig. 2. In HAK-1B cell line (Fig. 2A), significant difference in the relative viable cell number was observed between combination group and sorafenib or PEG-IFN-α2b alone groups, additionally, CI in all combination of PEG-IFN-α2b and sorafenib was <0.9. The CI was 0.879 in the combination of 2,000 IU/ml of PEG-IFN-α2b and 1.25 *μ*M of sorafenib, 0.667 in 4,000 IU/ml of PEG-IFN-α2b and 2.5 *μ*M of sorafenib, and 0.842 in 8,000 IU/ml of PEG-IFN-α2b and 5.0 *μ*M of sorafenib. According to the definition of the CI, these results indicate that a combination of PEG-IFN-α2b and sorafenib may produce a synergistic growth inhibitory effect in HAK-1B cell line. In KIM-1 cell line (Fig. 2B), there was also a significant difference in the relative viable cell numbers between combination group and monotherapy groups. The CI was 0.912 in the combination of 2,000 IU/ml of PEG-IFN-α2b and 1.25 *μ*M of sorafenib, 0.992 in 4,000 IU/ml of PEG-IFN-α2b and 2.5 *μ*M of sorafenib, and 0.823 in 8,000 IU/ml of PEG-IFN-α2b and 5.0 M of sorafenib. These results indicate that combination therapy may produce an additive or synergistic growth inhibitory effect in KIM-1 cell line.

**Figure 3:**
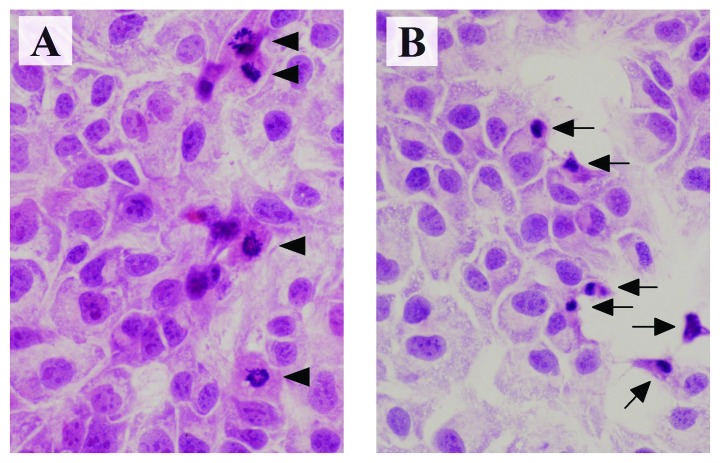
Photomicrograph of HAK-1B cells cultured for 72 h on Lab-Tek Chamber slide. (A) Without sorafenib in culture medium. Some mitotic figures were noted (arrowheads). (B) With 1.25 *μ*M of sorafenib in culture medium. There were some apoptotic cells characterized by cytoplasmic shrinkage and nuclear chromatin condensation (arrows).

Morphologically, HAK-1B cells showed characteristic features of apoptosis, such as cytoplasmic shrinkage and nuclear chromatin condensation at 72 h after adding 1.25 *μ*M of sorafenib (Fig. 3).

**Figure 4:**
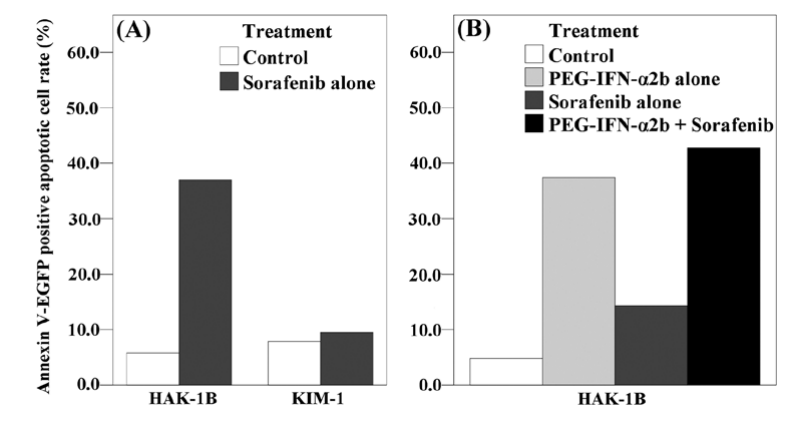
Quantitative analysis of Annexin V-EGFP positive apoptotic cells. (A) Apoptosis of HAK-1B or KIM-1 cells induced by 2 *μ*M of sorafenib. (B) Apoptosis of HAK-1B cells induced by 2,000 IU/ml of PEG-IFN-α2b and/or 1.25 M of sorafenib. Representative data of three independent experiments are shown.

The rate of Annexin V-EGFP positive apoptotic cells was increased by adding 2 *μ*M of sorafenib in HAK-1B cells (5.8% of the control and 37.8% of the sorafenib). In KIM-1 cells, however, the increase was relatively small (7.9% of the control and 9.5% of the sorafenib) (Fig. 4A). In another setting, the combination group with PEG-IFN-α2b showed higher rate of apoptosis than control or monotherapy groups in HAK-1B (4.8% of control, 37.4% of the PEG-IFN-α2b, 14.3% of the sorafenib, 42.8% of the combination) (Fig. 4B).

### Effects of sorafenib and/or PEG-IFN-α2b on HAK-1B or KIM-1 cell proliferation in nude mice

**Figure 5:**
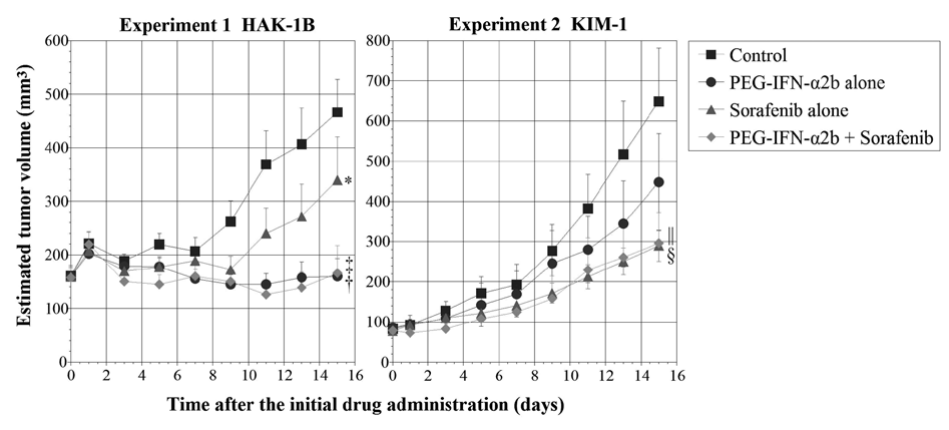
Chronological changes on the estimated volume of HAK-1B (Experiment 1) or KIM-1 (Experiment 2) tumor that was developed subcutaneously on nude mice. The PEG-IFN-α2b alone group (•) received subcutaneous injection of 1,920 IU twice a week for 14 days. The sorafenib alone group (▴) received 10 mg/kg/mouse/day orally every day for 14 days. The PEG-IFN-α2b + sorafenib group (♦) received 1,920 IU of PEG-IFN-α2b twice a week and 10 mg/kg of sorafenib every day for 14 days. The control group (▪) received subcutaneous injection of 0.1 ml of medium twice a week and 0.2 ml of Cremophor EL/ethanol/water (12.5/12.5/75). The values represent mean ± SE. ^*^P<0.05 vs. control. ^†^P<0.0001 vs. control, P<0.005 vs. sorafenib alone. ^‡^P<0.0001 vs. control, P<0.001 vs. sorafenib alone. ^§^P<0.001 vs. control. ^‖^P<0.0001 vs. control.

Chronological changes in estimated tumor volume after subcutaneous injection of cultured HAK-1B cells or KIM-1 cells to nude mice are summarized in Fig. 5. The actual tumor weights at the time of sacrifice are shown in Table I. In the experiment of HAK-1B tumors, the tumor volume of mice receiving PEG-IFN-α2b, sorafenib, and sorafenib+PEG-IFN-α2b was 34, 73 and 36%, respectively, of the control volume and the tumor weight was 23, 71 and 34%, respectively, of the control weight. Statistically, there were significant differences both in tumor volume and weight between the control group and the PEG-IFN-α2b alone group (P<0.0001 vs. control in tumor volume, P<0.0001 vs. control in tumor weight) or the combination group (P<0.0001 vs. control in tumor volume, P<0.001 vs. control in tumor weight) and between the sorafenib alone group and the PEG-IFN-α2b alone group (P<0.005 vs. sorafenib alone in tumor volume, P<0.05 vs. sorafenib alone in tumor weight). Although there was a significant difference between the sorafenib alone group and the combination group in tumor volume (P<0.001), this was not the case in the actual tumor weight (P=0.099). In the experiment of KIM-1 tumors, the tumor volume of mice receiving PEG-IFN-α2b, sorafenib, and sorafenib+PEG-IFN-α2b was 69, 45 and 46%, respectively, of the control volume and the tumor weight was 75, 41 and 37%, respectively, of the control weight. Statistically, there were significant differences in both tumor volume and weight between the control and the sorafenib alone group (P<0.0001 vs. control in tumor volume, P<0.05 vs. control in tumor weight) or the combination group (P<0.001 vs. control in tumor volume, P<0.05 vs. control in tumor weight).

The results of immunohistochemical examination are summarized in Table II. The significant decrease of MVD and increase of apoptotic cells were observed in the sorafenib group (P<0.0005 and 0.05 respectively vs. control in HAK-1B, P<0.05 and 0.05 respectively vs. control in KIM-1) and the combination group (P<0.05 and 0.05 respectively vs. control in HAK-1B, P<0.05 and 0.05, respectively, vs. control in KIM-1) compared to the control group in both HAK-1B and KIM-1 tumors, although there was no significant difference between the combination group and monotherapy groups. Ki67 labeling index was significantly lower in the combination group (P<0.005 vs. control, P<0.05 vs. PEG-IFN-α2b group) than in the control group or the PEG-IFN-α2b group only in KIM-1.

## Discussion

In this study, we showed the synergistic effect of sorafenib and PEG-IFN-α2b on HAK-1B cells *in vitro*. We previou sly reported that PEG-IFN-α2b induced apoptosis on both HAK-1B and KIM-1 cells *in vitro*(14). We found that sorafenib also induced apoptosis on HAK-1B *in vitro*. On the other hand, the increase of apoptotic cells was not clearly observed on KIM-1 cells in spite of the fact that the proliferation of KIM-1 cells was inhibited by sorafenib in MTT assay. A possible explanation is that cell proliferation might be inhibited by other antiproliferative mechanisms. The blockade of Raf signaling which is the main effect of sorafenib can lead to the repression of transforming growth factor α-epidermal growth factor receptor autocrine loops of tumor cells (5). Such a mechanism could have inhibited the growth of KIM-1 cells. In addition, a limitation of *in vitro* study is that we are not able to assess the indirect anti-angiogenic effect against endothelial cells.

In the *in vivo* study, there was a significant reduction of tumor volume and weight in the combination group on both HAK-1B and KIM-1 tumors compared with the control group. However, there was no significant difference between the combination and the monotherapy groups, and it seemed that HAK-1B tumors were sensitive to PEG-IFN-α2b and KIM-1 tumors to sorafenib. Only in KIM-1 tumors that might be sensitive to sorafenib, Ki67 labeling index was lower in the combination group than in the control group. Recently Wang *et al*(25) reported that combination therapy of sorafenib with recombinant human INF-α2a was effective *in vitro* and *in vivo* on two HCC cell lines, Huh-7 and Sk-Hep-1. In their study, the significant differences between combination and monotherapy groups were clearly observed. This partial difference might be due to the different experimental settings, such as different cell lines and different dose of drugs. One of the greatest differences, we surmise, is the site of IFN administration. They injected IFN directly into subcutaneous tumors, whereas we did subcutaneously but not into the tumors.

Since sorafenib is a multikinase inhibitor, it is considered that sorafenib has both direct antiproliferative effect due to the blockade of Raf kinase on tumor cells themselves and indirect effect due to the blockade of receptor tyrosine kinases, such as VEGFR-2, on endothelial cells followed by the inhibition of angiogenesis (5). Therefore we also evaluated MVD of xenografts and confirmed the significant decrease of MVD in the sorafenib alone and the combination group in both HAK-1B and KIM-1 tumors. It has been repeatedly shown that IFN suppresses the growth of various types of human tumors that were transplanted into mice through the anti-angiogenic effect. Tedjarati *et al*(26) reported that the subcutaneous injection of 7,000 IU per week of PEG-IFN-α2b into nude mice bearing human ovarian cancer cells induced a significant decrease of CD31-positive endothelial cells and Huang *et al*(27) showed similar results with the subcutaneous injection of 70,000 IU per week of PEG-IFN-α2b on human prostate cancer cells. PEG-IFN-α2b administered at higher or lower doses was less effective. In our current study, however, there was no significant decrease of MVD in the PEG-IFN-α2b group compared with the control group. Moreover, in our previous report, the decrease of artery-like blood vessels was not observed in the same HAK-1B tumors by the administration of PEG-IFN-α2b at either higher or lower doses (14).

Another notable finding regarding the MVD in this study is the discrepancy between MVD and tumor weight or size. Interestingly, the reduction of tumor weight and size was not so much in sorafenib monotherapy group in HAK-1B tumors despite the most prominent decrease of MVD was observed in this group. On the other hand, there was a significant reduction of tumor weight and size in PEG-IFN-α2b alone group in HAK-1B, although this group did not show any significant decrease of MVD. This result supports our previous findings in which we showed there was no relationship between tumor shrinkage and the number of artery-like blood vessels in HAK-1B tumors after the administration of the various concentration of PEG-IFN-α2b (14). Hlatky *et al*(28) mentioned in their review article that the efficacy of anti-angiogenic agents cannot be simply visualized by alterations in microvessel density during treatment because of the difference of the tightness of the coupling between vessel drop-out and tumor-cell drop-out after the treatment. In addition, Yao *et al*(29) recently reported that the expression of VEGFR-1 in tumor cells which is normally expressed specifically in endothelial cells were strongly associated with anti-PlGF antibody efficacy, but not with anti-angiogenesis. More studies are needed to investigate new approaches to assess the efficacy of anti-angiogenic drugs *in vivo* and molecular mechanisms of their action of ‘anti-angiogenic’ drugs.

In conclusion, we demonstrated the synergistic antiproliferative effect of combination therapy on HAK-1B cells *in vitro*. Although, *in vivo* the synergistic effects of the combination therapy were not clearly observed, the combination therapy induced nearly maximal antitumor effects, independent of the HCC cell sensitivity to antitumor effects of single therapy with either PEG-IFN-α2b or sorafenib. These findings suggest that PEG-IFN-α2b might be a promising candidate for use in combination therapy with sorafenib and warrant further investigation.

## Figures and Tables

**Table I:** The weight of subcutaneous tumors of HAK-1B cells or KIM-1 cells in nude mice at sacrifice.

	Tumor weight (g)	
Treatment group	HAK-1B	KIM-1
Control	0.333±0.03	0.504±0.17
PEG-IFN-α2b alone	0.078±0.02^a^	0.379±0.18
Sorafenib alone	0.236±0.06	0.206±0.04^c^
PEG-IFN-α2b + sorafenib	0.113±0.04^b^	0.185±0.12^c^

Tumor weight represents mean ± SE (g).

a P<0.0001 vs. control, P<0.05 vs. sorafenib alone.

b P<0.001 vs. control.

c P<0.05 vs. control.

**Table II:** MVD and the ratio of apoptotic cells and Ki67 positive cells in human HCC tumors subcutaneously transplanted in nude mice.

Cell line	Treatment group	MVD	Apoptotic cells	Ki67 positive cells
HAK-1B	Control	100.8±7.7	3.8±0.4	36.8±2.0
	Peg-IFN-α2b alone	114.9±16.7	4.4±0.4	37.5±4.6
	Sorafenib alone	53.8±4.3^a^	6.7±1.3^b^	38.3±2.0
	Peg-IFN-α2b + soragfenib	69.4±10.1^b^	5.6±1.3^b^	35.3±2.2
KIM-1	Control	125.9±16.2	4.6±0.4	6.7±0.2
	Peg-IFN-α2b alone	97.4±10.4	5.1±0.4	7.5±0.8
	Sorafenib alone	85.1±6.6^b^	6.5±0.7^b^	5.7±0.4
	Peg-IFN-α2b + soragfenib	79.0±7.2^b^	6.3±0.6^b^	4.6±0.5^c^

Scores represent mean ± SE.

a P<0.0005 vs. control, P<0.01 vs. Peg-IFN-α2b alone.

b P<0.05 vs. control.

c P<0.005 vs. control, P<0.05 vs. Peg-IFN-α2b alone.
